# Managing noradrenaline after traumatic brain injury

**DOI:** 10.1002/ctm2.1562

**Published:** 2024-01-27

**Authors:** Rashad Hussain, Maiken Nedergaard

**Affiliations:** ^1^ Center for Translational Neuromedicine University of Rochester Rochester New York USA; ^2^ Center for Translational Neuroscience University of Copenhagen Copenhagen Denmark

1

Traumatic brain injury (TBI) affects roughly 55−74 million people per year worldwide and is a leading cause of death and disability in young adults. As such, TBI has profound and long‐lasting effects on individuals, families and the healthcare system. Treatment is particularly challenging due to a complex interplay of causative factors, aetiology and management. A particularly dire manifestation of TBI is cerebral oedema, which increases the risk of death by 10‐fold and the chances of disability in patients who survive the initial injury. Despite the fact that the treatment of TBI patients is a complex phenomenon, noradrenaline (NA) has been the drug of choice in trauma clinics to counteract hypotension and maintenance of cerebral perfusion pressure. NA's ability to constrict blood vessels results in increased vascular resistance, which helps to elevate blood pressure and maintain adequate perfusion to vital organs.

Paradoxically, clinical evidence suggests a dramatic upregulation of NA in plasma and cerebral spinal fluid (CSF), immediately after brain injury.^1^, ^2^ The two possible sources of NA are the central brainstem noradrenergic nuclei and peripheral adrenal medulla via release into bloodstream. Plasma NA increases to very high concentrations after multiple types of injury and is a likely predominant source of post‐traumatic NA.

In a newly published study,^3^ we interrogated post‐TBI cerebral oedema using the ‘Hit‐and‐Run’ mouse model. A substantial increase in brain water content was observed; initially in the ipsilateral hemisphere and subsequently in the contralateral hemisphere. Microdialysis of the extracellular fluid from the contralateral hemisphere showed that NA widely fluctuates and peaks at 2 hours post‐injury. Concurrently, the glymphatic system which is the brain's waste clearance pathway involving the transport of CSF through the brain's perivascular space to facilitate the removal of solutes and excess fluids shuts down and fluid starts accumulating within the brain. This signifies that cerebral oedema is not a result of increased CSF influx, but rather of the ability of the injured brain to export the excess fluid. The major observation was that the pan‐adrenergic blockage, based on the administration of a cocktail of α1, α2 and β receptor antagonists reduced or eliminated the acute oedema bilaterally. Remarkably, the pan‐adrenergic receptor blockage also enhanced neurological functionality, as evidenced by improved performance in various behavioural assays, including the Morris water maze test.

The study also delved into the role of cervical lymphatic drainage in TBI, revealing its impairment due to the excessive increase in NA. Pan‐adrenergic blockage was found to improve, significantly, the function of cervical lymphatic vessels draining CSF and interstitial fluid out of central nervous system (CNS), as evidenced by increased lymph vessel contractility and enhanced velocity of lymph efflux. The impairment of the glymphatic/lymphatic system results not only in oedema but also leads to the accumulation of metabolic waste and cellular debris, which become ensnared within the brain's interstitial spaces (as illustrated in Figure 1). In turn, the entrapment of cellular debris initiates a vicious cycle of neuro‐inflammation and neural tissue degeneration. Intriguingly, we visualised cellular debris being washed out of the post‐traumatic brain via cervical lymphatic vessels using high‐resolution two‐photon microscopy after administering the cocktail of noradrenergic receptor blockers. This suggests that the efficacy of pan‐adrenergic inhibitors extends beyond mere oedema reduction to facilitating the clearance of cellular debris and enhancing overall glymphatic/lymphatic function. In support of this finding, other studies have reported that ultraviolet irradiation of meningeal lymphatic vessels^4^ or physical ligation of cervical lymphatic vessels^5^ exacerbate cognitive impairment in murine Alzheimer models, highlighting the critical role of lymphatic drainage in brain health and function.

**FIGURE 1:**
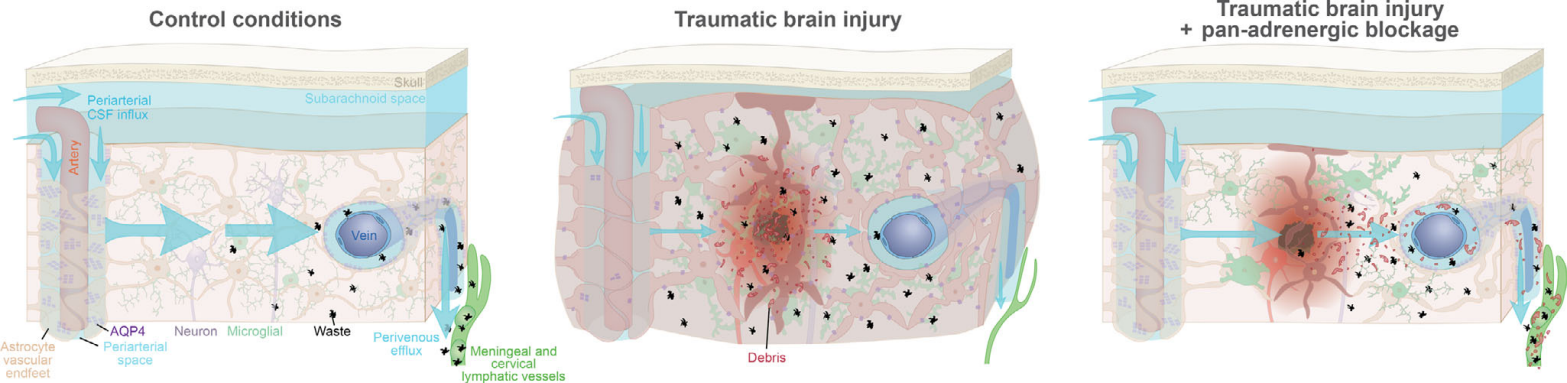
Illustration of glymphatic impairment in traumatic brain injury (TBI) and how noradrenergic inhibition helps in removing cellular debris. (Left) The glymphatic system in a healthy brain; periarterial CSF influx along the blood vessels (blue arrows point the direction), water channel AQP4 are distributed along astrocyte endfeet, the efflux of metabolic waste into perivenous spaces and meningeal lymphatic vessels draining into cervical lymphatic vessels. (Middle) Disrupted cerebral physiology and glymphatic flow following TBI; there is a marked increase in waste products and cellular debris, indicated by the red and black symbols, and disrupted CSF flow and waste clearance pathways, emphasising the effects of TBI on brain homeostasis. Neuronal swelling, depolarisation of AQP4 and glial hypertrophy are also shown. (Right) Pan‐adrenergic blockage restores the glymphatic flow and consequently reduces the accumulation of waste and debris, thus suggesting a therapeutic intervention to reduce oedema and buildup of waste restore, and neurodegeneration in the long run.

Sympathetic overactivation, which can persist for weeks and even months, in the aftermath of TBI has been a focus in several clinical studies but the absence/lack of mechanistic understanding hindered a change in the clinical practice. NA released by the brain stem modulates the sympathetic nervous functions, which include maintaining the cardiovascular rhythms by acting on α1, α2 and β receptors, classified as G‐protein coupled receptors. The signalling cascade typically involves cyclic adenosine monophosphate (cAMP) and protein kinase A (PKA) upregulation which together with other factors are responsible for the cytosolic Ca^++^ increase that drives cardiac contractility. Moderate to severe TBI specifically induces tachycardia, characterised by an elevated heart rate along with cardiac arrhythmias, such as arterial fibrillation,^6^ as a direct consequence of NA surges. Emerging clinical evidences show that NA administration can lead to an increase in intracranial pressure, cerebral ischaemia and stroke with minimal to no improvement in long‐term recovery and functional outcome.^7^ The sympathetic storm^1^, ^2^ that ensues TBI can manifest in an array of additional symptoms including hyperthermia, tachypnea, diaphoresis and dystonia—which encompasses hypertonia or spasticity—and can extend to motor anomalies such as extensor/flexion posturing,^8^ each of these symptoms has a unique receptor‐mediated cascade, discussion is beyond the scope of this commentary.

Despite the beneficial effects of NA on cerebral perfusion pressure, NA treatment increased the interleukin 6 after experimental TBI, beyond the spike.^9^ Another harmful consequence of NA therapy after TBI is a reduction in creatinine clearances both during NA treatment and following its discontinuation.^10^ Platelet activation studies suggest that clinically infused NA might influence platelets, possibly promoting microthrombosis formation. Particularly in the second week post‐injury TBI patient's platelets exhibited hyper‐susceptibility to NA exposure which coincided with increased intracranial pressure and reduced saturation of blood within the jugular vein.^11^ A meta‐analysis (15 studies, 12 721 patients) suggests that administration of β‐blockers after TBI was associated with a significant reduction in adjusted in‐hospital mortality, improvement of functional outcome, and minimal adverse effects.^12^ A pilot study suggests a prophylactic role of α1 blockage in treating posttraumatic headache.^13^ Similarly, its use alleviates delayed post‐TBI symptoms insomnia, and nightmares after trauma.^14^ Early treatment with α2 receptor antagonists, which are commonly used to reverse sedative and analgesic effects of other drugs, may reduce the chances of post‐traumatic seizures.^15^

In short, many other studies point out the beneficial effect of noradrenergic inhibition after exposure to traumatic head injury but the mechanistic insights were missing. Our preclinical study provides mechanistic insight pointing towards the beneficial effects of pan‐adrenergic blockage is a result of the reduction of cerebral oedema and the timely removal of cellular debris from the brain via glymphatic/lymphatic restoration.

## AUTHOR CONTRIBUTIONS

Rashad Hussain and Maiken Nedergaard wrote and edited the commentary.

## CONFLICT OF INTEREST STATEMENT

The authors declare they have no conflicts of interest. Maiken Nedergaard is a paid consultant of CNS2 for unrelated work.
